# Presumed pituitary apoplexy in 26 dogs: Clinical findings, treatments, and outcomes

**DOI:** 10.1111/jvim.16703

**Published:** 2023-04-21

**Authors:** Christian W. Woelfel, Christopher L. Mariani, Michael W. Nolan, Erin K. Keenihan, Sophia P. Topulos, Peter J. Early, Karen R. Muñana, Sarah E. Musulin, Natasha J. Olby

**Affiliations:** ^1^ Veterinary Hospital, College of Veterinary Medicine North Carolina State University Raleigh North Carolina USA; ^2^ Department of Clinical Sciences, College of Veterinary Medicine North Carolina State University Raleigh North Carolina USA; ^3^ Comparative Neuroimmunology and Neuro‐Oncology Laboratory North Carolina State University Raleigh North Carolina USA; ^4^ Comparative Medicine Institute North Carolina State University Raleigh North Carolina USA; ^5^ Department of Molecular Biomedical Sciences, College of Veterinary Medicine North Carolina State University Raleigh North Carolina USA; ^6^ Present address: Garden State Veterinary Specialists Tinton Falls New Jersey USA; ^7^ Present address: Department of Small Animal Soft Tissue Surgery, College of Veterinary Medicine University of Illinois Urbana‐Champaign Urbana IL

**Keywords:** adenoma, carcinoma, endocrionopathy, hemorrhage, magnetic resonance imaging, suprasellar, survival

## Abstract

**Background:**

Pituitary apoplexy refers to hemorrhage or infarction within the pituitary gland resulting in acute neurological abnormalities. This condition is poorly described in dogs.

**Objectives:**

To document presenting complaints, examination findings, endocrinopathies, magnetic resonance imaging (MRI), treatments, and outcomes of dogs with pituitary apoplexy.

**Animals:**

Twenty‐six client‐owned dogs with acute onset of neurological dysfunction.

**Methods:**

Retrospective case series. Dogs were diagnosed with pituitary apoplexy if MRI or histopathology documented an intrasellar or suprasellar mass with evidence of hemorrhage or infarction in conjunction with acute neurological dysfunction. Clinical information was obtained from medical records and imaging reports.

**Results:**

Common presenting complaints included altered mentation (16/26, 62%) and gastrointestinal dysfunction (14/26, 54%). Gait or posture changes (22/26, 85%), mentation changes (18/26, 69%), cranial neuropathies (17/26, 65%), cervical or head hyperpathia (12/26, 46%), and hyperthermia (8/26, 31%) were the most frequent exam findings. Ten dogs (38%) lacked evidence of an endocrinopathy before presentation. Common MRI findings included T1‐weighted hypo‐ to isointensity of the hemorrhagic lesion (21/25, 84%), peripheral enhancement of the pituitary mass lesion (15/25, 60%), brain herniation (14/25, 56%), and obstructive hydrocephalus (13/25, 52%). Fifteen dogs (58%) survived to hospital discharge. Seven of these dogs received medical management alone (median survival 143 days; range, 7‐641 days) and 8 received medications and radiation therapy (median survival 973 days; range, 41‐1719 days).

**Conclusions and Clinical Importance:**

Dogs with pituitary apoplexy present with a variety of acute signs of neurological disease and inconsistent endocrine dysfunction. Dogs that survive to discharge can have a favorable outcome.

**Abbreviations**
ADC: apparent diffusion coefficient
CT: computed tomography
DWI: diffusion‐weighted imaging
FLAIR: fluid attenuated inversion recovery
GRE: gradient echo
MGCS: modified Glasgow coma score (MGCS)
MRI: magnetic resonance imaging
PD: proton density
SWI: susceptibility‐weighted imaging
T1W: T1‐weighted
T2W: T2‐weighted

## 1 INTRODUCTION

Pituitary apoplexy is a well‐described clinical syndrome in humans, which results from acute hemorrhage or infarction within the pituitary gland.^1^ Typically, this condition occurs with a preexisting insidious pituitary adenoma^2^, ^3^, ^4^ and affects an estimated 2.0%‐14.1% of patients with such tumors.^2^, ^5^, ^6^ Human patients experience a constellation of acute signs of neurological and endocrine disease, including severe headache, nausea, vomiting, visual impairment, altered levels of consciousness, cranial nerve palsies, hormonal disturbances, and sudden death.^2^, ^7^ Several predisposing factors have been identified in people, including pregnancy, anticoagulant therapy, thrombocytopenia, and gonadotropin‐releasing hormone treatment.^5^ The etiology of pituitary apoplexy, however, remains elusive, with explanatory theories including tumor growth outstripping its vascular supply and tumor‐induced compromise and compression of the thin intrasellar vascular network.^2^, ^3^, ^8^ Recommended treatment is controversial and there are varied opinions on transsphenoidal surgical decompression vs conservative management based on the severity of clinical signs.^9^ The case fatality rate of pituitary apoplexy in humans ranges from 1.6% to 1.9%, with most patients needing long‐term hormonal replacement therapy to manage secondary adrenal insufficiency or central diabetes insipidus.^4^, ^5^, ^10^, ^11^

By comparison, there is a paucity of literature on pituitary apoplexy in dogs and cats. Four case reports with a combined total of 6 dogs described acute onsets of clinical signs, including vomiting, altered consciousness, behavioral changes, vision loss, seizures, internal ophthalmoplegia and collapse.^12^, ^13^, ^14^ A recent case series of 19 dogs (including 2 of the previously reported 6 dogs) described mainly altered mentation, vestibular dysfunction and seizures.^15^ While computed tomography, histopathology, and postmortem findings are described in this literature, in addition to magnetic resonance imaging (MRI) findings of pituitary apoplexy in a cat,^16^ published MRI findings in dogs with pituitary apoplexy appear to be limited to 8 cases.^14^, ^15^ Treatment options and outcome data are also limited in the veterinary literature, as many dogs are euthanized at the time of diagnosis or fail to survive to hospital discharge.^13^, ^15^

The objective of our study was to augment preexisting literature on pituitary apoplexy in dogs by describing a larger cohort of dogs, with a focus on historical comorbidities and endocrinopathies, presenting complaints from owners, physical as well as neurological exam abnormalities, and clinicopathologic abnormalities. Additional goals were to document MRI findings and to detail attempted therapies and outcomes for pituitary apoplexy in dogs.

## 2 MATERIALS AND METHODS

### 2.1 Case selection

Dogs diagnosed with pituitary apoplexy at NC State University between July 2008 and April 2021 were identified retrospectively. Medical record imaging reports and discharge summaries were searched, using combinations of the following terms: pituitary, apoplexy, hemorrhage, infarct, bleed, macroadenoma, tumor, lesion, and mass. Dogs were included if they had an acute onset of obvious signs of neurological disease (eg, seizures, change in consciousness, circling) and evidence of pituitary hemorrhage or infarction on MRI or postmortem examination. Imaging studies were generated with either a 1.5T or 3.0T system (MAGNETOM Symphony and MAGNETOM Skyra, respectively, Siemens Medical Solutions, USA, Malvern, Pennsylvania). Images of the brain were reviewed using transverse and sagittal sequences (T1‐weighted [T1W] or T1W fluid attenuated inversion recovery [FLAIR] pre‐ and post‐contrast, T2‐weighted [T2W], gradient echo [GRE]/T2* or susceptibility‐weighted imaging [SWI], proton density [PD], T2W FLAIR, diffusion‐weighted imaging [DWI], trace, and apparent diffusion coefficient [ADC] map) to confirm the presence of an intrasellar or suprasellar pituitary mass lesion with evidence of hemorrhage or infarction. MRI studies were evaluated by a board‐certified radiologist at the time of acquisition and were reviewed by 1 of the authors (Christian W. Woelfel). Neurological examinations were performed by a neurology resident or board‐certified neurologist. Dogs with vague or chronically progressive signs of neurological disease (defined as a duration >1 week), or absence of neurological abnormalities on examination were excluded.

### 2.2 Clinical information

Dog signalments, histories, presence of preexisting endocrinopathies or other diseases, physical and neurological exam abnormalities, diagnostic imaging findings, postmortem pathology, treatments initiated, and outcomes were obtained from the medical record. A modified Glasgow coma score (MGCS) was determined retrospectively for each dog after review of the medical record.^17^, ^18^ Additionally, the results of clinical pathology testing, including complete blood counts, serum chemistries, urinalyses, coagulation testing, endocrine evaluation, and other diagnostic tests were recorded. Methods of follow‐up included recheck examinations at our hospital or with referring veterinarians, and telephone interviews with owners. Survival was determined as the time from diagnosis until death from any cause.

### 2.3 Statistical analyses

Categorical data were reported as frequency of occurrence. Ordinal data (MGCS) were reported as medians with ranges. Continuous data were listed as means with standard deviations and ranges, after D'Agostino and Pearson testing showed these distributions to be normal. The MGCS between groups were compared with a Mann‐Whitney test. Associations between clinical signs, brain herniation, hyperosmolar treatment and survival to hospital discharge were analyzed using a Fisher's exact test. Overall survival time was described using the Kaplan‐Meier method and comparisons between groups were made using a log‐rank test. All analyses were performed with Prism (version 9.0.0, Graphpad Software, La Jolla, California) and a *P* value <.05 was considered significant.

## 3 RESULTS

Thirty dogs were identified to have advanced imaging or postmortem findings consistent with pituitary hemorrhage or infarction. Four dogs were excluded because of chronicity of signs or lack of neurological abnormalities, leaving 26 dogs that fulfilled our criteria for pituitary apoplexy.

There were 6 Labrador Retrievers, 5 mixed breed dogs, 3 Australian Shepherds, 2 Boston Terriers, 2 Golden Retrievers, 2 American Staffordshire Terriers, and 1 each of the following breeds: Siberian Husky, Miniature Pinscher, Catahoula Leopard Dog, Boxer, Beagle, and English Bulldog. The mean age was 8.6 ± 2.3 years (range, 4‐14 years) and the mean weight was 22.5 ± 9.0 kg (range, 4.4‐39.8 kg). There were 15 castrated male, 3 intact male, and 8 spayed female dogs.

**FIGURE 1:**
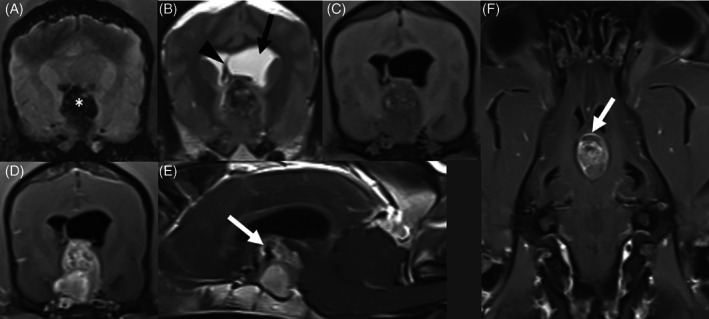
Magnetic resonance imaging of pituitary apoplexy in the same dog. (A) Transverse susceptibility‐weighted imaging (SWI) reveals a large suprasellar blooming artifact (marked with white asterisk). (B) Transverse T2‐weighted (T2W) image revealing an ovoid suprasellar mass which is heterogeneously T2 hypointense with internal T2 hyperintense foci relative to surrounding gray matter. Obstructive hydrocephalus of the left lateral ventricle is marked with the black arrow, with deviation of the septum pellucidum (black arrowhead), consistent with a falcine shift. (C) Transverse T1‐weighted (T1W) image heterogeneously T1 hypointense with internal T1 hyperintense foci relative to surrounding gray matter. (D‐F) Respectively, transverse, sagittal, and dorsal T1W images after contrast administration, showing strong contrast‐enhancement with some areas of ring‐enhancement (white arrows). There is flattening of the rostral cerebellum and compression of the rostral colliculi, consistent with transtentorial herniation.

Summary data on previously diagnosed endocrinopathies, owners' presenting complaints, and exam abnormalities are presented in Table 1. Twelve dogs (46%) had lateralizing neurological abnormalities on examination (eg, circling, head tilt, unilateral menace deficit). The median MGCS for the entire cohort was 16 (9–18). The mean indirect systolic blood pressure on presentation was available for 22/26 dogs and was 142.2 ± 26.1 mm Hg (range, 80‐200 mm Hg). Clinicopathologic and MRI findings are documented in Table 2. All MR images showed a large, well‐defined, ovoid, suprasellar, extra‐axial, contrast‐enhancing mass lesion causing compression of the diencephalon, with intralesional susceptibility artifact on T2* or SWI sequences (Figure 1), indicative of hemorrhage. One dog also had susceptibility artifacts within the lateral, third, and fourth ventricles, suggesting hemorrhage within the ventricular system. Six dogs had diffusion‐weighted imaging studies, which were not supportive of pituitary infarction (Table 2). Another dog had a strongly contrast‐enhancing, extra‐axial, plaque‐like mass lesion, in addition to the pituitary tumor, that was causing mild ventral compression of the medulla oblongata and was most consistent with a meningioma. Seven dogs had signs consistent with vestibular dysfunction, of which 4 had no evidence of brain herniation on MRI, 2 had transtentorial herniation and 1 had subfalcine herniation. Seven dogs had transtentorial and 3 dogs had subfalcine herniation without signs of vestibular dysfunction. The dog with the suspected brainstem meningioma did not have vestibular signs. There were no significant associations detected between vomiting, ptyalism or vestibular dysfunction and brain herniation (Table 3). Endocrine testing included ACTH stimulation (4 dogs), baseline cortisol (4), total thyroxine (T_4_) and thyroid stimulating hormone (4), total T_4_ (3), low‐dose dexamethasone suppression (1), ACTH concentration (1), total and free T_4_ (1), and a thyroid panel with total and free T_4_, thyroid stimulating hormone, triiodothyronine (T_3_) and autoantibodies to T_4_ and T_3_ (1). Analysis of cerebrospinal fluid collected from the cerebellomedullary cistern was performed in 5 dogs. All analyses were abnormal and showed pleocytosis in 4 dogs, albuminocytologic dissociation in 1 dog and evidence of erythrophagocytosis in 2 dogs (Table S1).

**TABLE 1:** Presenting complaints, histories, and examination findings of dogs with pituitary apoplexy.

	N = 26
Historical endocrinopathy	
*Endocrinopathies* ^a^	13 (50%)
Hyperadrenocorticism	9 (35%)
Hypothyroidism	6 (23%)
Clinical signs suggestive of endocrinopathy without workup^b^	3 (12%)
Absence of/subclinical endocrinopathy	10 (38%)
Presenting complaints	
Altered mentation/head pressing	16 (62%)
Signs of gastrointestinal dysfunction^c^	14 (54%)
Unable to walk/collapse	9 (35%)
Ataxia	9 (35%)
Restlessness/anxiety/pacing	9 (35%)
Seizure	5 (19%)
House soiling/inappropriate urination	5 (19%)
Posture change^d^	3 (12%)
Visual impairment^e^	3 (12%)
Exam abnormalities	
Hyperthermia^f^	8 (31%)
Bradycardia^g^	4 (15%)
Mentation change	18 (69%)
Cranial neuropathies	17 (65%)
Strabismus	7 (27%)
Anisocoria/mydriasis/miosis	7 (27%)
Head tilt	4 (15%)
External ophthalmoparesis/plegia^h^	3 (12%)
Spontaneous nystagmus	2 (8%)
Gait/posture change	22 (85%)
Ambulatory	14 (54%)
Circling	6 (23%)
Paresis	3 (12%)
Ataxia	9 (35%)
Vestibular	6 (23%)
Proprioceptive	3 (12%)
Low head carriage/kyphosis	2 (8%)
Nonambulatory	8 (31%)
Decerebrate posturing	2 (8%)
Cervical/head hyperpathia	12 (46%)

^a^ Two dogs had both hypothyroidism and hyperadrenocorticism.

^b^ Clinical signs included polyuria and polydipsia, poor haircoat/symmetrical trunk alopecia, or pot‐bellied appearance of at least 1 month before acute neurologic signs, with no diagnostic workup previously performed by the referring veterinarian.

^c^ Signs of gastrointestinal dysfunction included acute vomiting, ptyalism, and hyporexia/anorexia.

^d^ Posture changes were consistent with either kyphosis or opisthotonos.

^e^ Visual impairment determined by walking into objects.

^f^ Defined as ≥102.5° Fahrenheit.

^g^ Defined as ≤100 beats per minute for dogs ≤10 kg and ≤60 beats per minute for dogs >10 kg.

^h^ One dog also had internal ophthalmoplegia, as evidenced by absent pupillary light reflex bilaterally.

**TABLE 2:** Clinicopathologic and magnetic resonance imaging findings in dogs with pituitary apoplexy.

Clinicopathologic findings	
Serum chemistry	N = 24
Hepatopathy^a^	11 (46%)
Azotemia	3 (13%)
Normal	11 (46%)
Complete blood count	N = 23
Stress leukogram	3 (13%)
Thrombocytopenia^b^	3 (13%)
Nonregenerative anemia	2 (9%)
Thrombocytosis^b^	1 (4%)
Normal	14 (61%)
Urinalysis^c^	N = 13
Inappropriate urine concentration	9 (69%)
Hyposthenuria	3 (23%)
Isosthenuria	2 (15%)
Minimally concentrated urine	4 (31%)
Proteinuria	3 (23%)
Normal	3 (23%)
Coagulation testing	N = 4
Mild hypocoagulability^d^	1 (25%)
Normal	3 (75%)

MRI findings	N = 25
Contrast‐enhancement	25 (100%)
Peripheral/ring‐enhancement^e^	18 (72%)
Heterogeneous	6 (28%)
Homogeneous	1 (4%)
Obstructive hydrocephalus	13 (52%)
Brain herniation^f^	12 (48%)
Transtentorial	9 (36%)
Subfalcine	4 (16%)
Intralesional susceptibility artifact on T2* or SWI imaging	25 (100%)
Intensity of hemorrhage relative to adjacent gray matter	
T1W appearance	
Hypointense to isointense	21 (84%)
Hyperintense to isointense	1 (4%)
Mixed hyper‐ and hypointense	3 (12%)
T2W appearance	
Hypointense to isointense	13 (52%)
Hyperintense to isointense	9 (36%)
Mixed hyper‐ and hypointense	3 (12%)
DWI/ADC appearance	6 (24%)
Hypointense for both	3 (50%)
Mixed, mostly hypointense for both	2 (33%)
Hyperintense for both	1 (17%)

Abbreviations: ADC, apparent diffusion coefficient; DWI, diffusion‐weighted imaging; MRI, magnetic resonance imaging; T1W, T1‐weighted; T2W, T2‐weighted.

^a^ Hepatopies include evidence of hepatocellular (alanine aminotransferase or aspartate aminotransferase) or cholestatic (gamma‐glutamyl transferase or alkaline phosphatase) dysfunction, or a combination of both.

^b^ Platelet count reference range 190 000‐468 000 platelets/μL. The 3 dogs represented had 61 000; 71 000; and 121 000 platelets/μL.

^c^ Urine specific gravities were defined as follows: >1.030: appropriately concentrated urine; 1.013‐1.029: minimally concentrated urine; 1.008‐1.012: isosthenuria, <1.008 hyposthenuria. Proteinuria defined as protein in the urine in the absence of a urinary tract infection or red blood cells. Only 1 patient under this definition had proteinuria confirmed with a urine protein‐creatinine ratio. Two dogs had both urine concentration abnormalities and proteinuria.

^d^ This dog had a partial thromboplastin time that was 1.3× the high end of the reference range.

^e^ Defined as contrast enhancement that is more pronounced for the periphery of the mass lesion as compared to the centrally.

^f^ One dog had both transtentorial and subfalcine herniation.

**TABLE 3:** Associations between clinical signs, brain herniation, hyperosmotic therapy, and outcome.

Condition		Outcome			*P* value^a^
		Yes	No	Total	
Brain herniation					
Vomiting or ptyalism	Yes	3	6	9	.43
	No	9	8	17	
Vestibular dysfunction	Yes	3	4	7	>.99
	No	9	10	19	
Survival					
Hyperthermia	Yes	3	5	8	.22
	No	12	6	18	
Vomiting or ptyalism	Yes	4	5	9	.42
	No	11	6	17	
Vestibular dysfunction	Yes	6	1	7	.18
	No	9	10	19	
Brain herniation	Yes	8	4	12	.45
	No	7	7	14	
Hyperosmotic agents	Yes	3	8	11	.02
	No	11	3	14	

^a^ Fisher's exact test.

**FIGURE 2:**
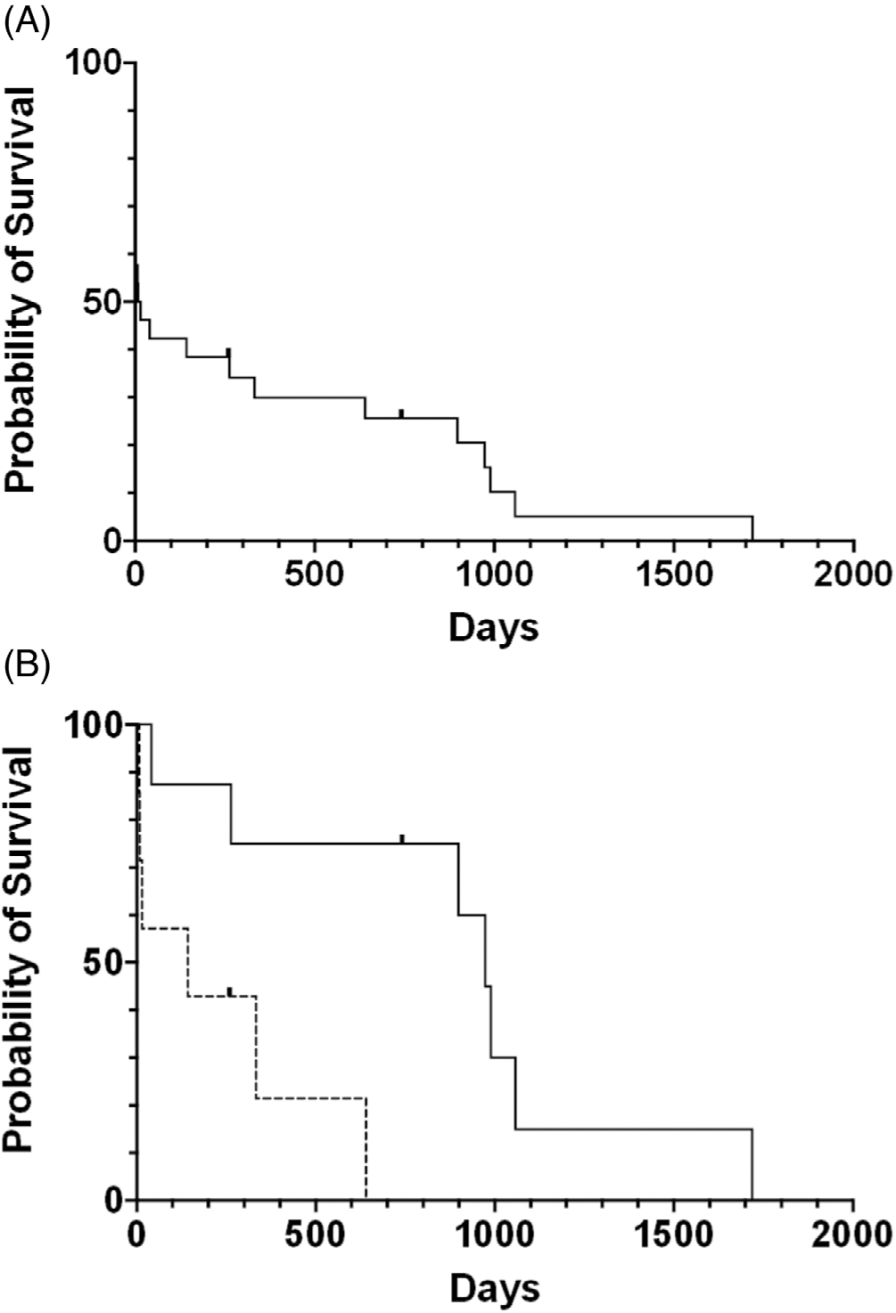
Survival curves for (A) the entire cohort of dogs with pituitary apoplexy and (B) those dogs that survived to hospital discharge. Dogs that received radiation therapy in addition to medical therapy (solid line) survived longer than those receiving medical therapy alone (dashed line, *P* = .008). Ticks represent dogs that were censored during analysis.

The median survival time for the entire cohort was 11.5 days (range, 1‐1719 days; Figure 2A). Eleven dogs (42%) were hospitalized for supportive care, including hyperosmotic agents, glucocorticoids, intravascular fluid therapy, and anticonvulsant medications before or after the MRI, but did not have a favorable response (eg, deterioration despite supportive care or inadequate improvement suitable for discharge as determined by the clinician or owner) and humane euthanasia during hospitalization was elected. The median MGCS of these dogs was 15 (9–17). One of these dogs had an MRI and a subsequent postmortem examination, that revealed a pituitary adenoma with intralesional hemorrhage. Another dog was euthanized without an MRI because of a stuporous mentation and a lack of response to mannitol and glucocorticoids, and was later confirmed to have a large pituitary carcinoma with acute hemorrhage and thalamic compression on postmortem examination. Necropsies were not performed in the remaining dogs.

The remaining 15 dogs (58%) survived to hospital discharge. The median MGCS of these dogs was 16 (10–18), which was significantly different from the MGCS of dogs not surviving to discharge (*P* = .014). We found no statistically significant associations between clinical signs or radiographic evidence of brain herniation and outcome (Table 3). Seven of these dogs (47%) received medications alone, including hyperosmotic agents, glucocorticoids, analgesic medications, proton pump inhibitors, and anticonvulsant medications. The median survival time of these medically managed dogs was 143 days (range, 7‐641 days). Dogs receiving hyperosmotic agents were less likely to survive to hospital discharge than dogs not receiving these medications (*P* = .02). Eight of the 15 dogs (53%) received radiation therapy in addition to medications. Among these dogs, there was no standard radiotherapeutic approach. All dogs were treated on a clinical linear accelerator, using 6 MV X‐rays. Seven dogs were treated with definitive‐intent protocols, which included 5 dogs receiving conventional full‐course fractionated radiotherapy, 1 dog receiving a 5‐fraction stereotactic radiotherapy protocol and 1 dog treated with single fraction stereotactic radiosurgery. The final dog was treated with a palliative‐intent protocol. Comprehensive dosimetric characterization of these plans^19^ is beyond the scope of this manuscript; however, additional treatment plan details are provided in the Supplemental Materials (Table S2). The median survival time of these 8 dogs was 973 days (range, 41‐1719 days), which was significantly longer than those treated solely with medical therapy (*P* = .008; Figure 2B). The dog with a survival of 41 days succumbed to splenic hemangiosarcoma, which was undetected at the time of the pituitary apoplexy diagnosis. One of these dogs needed long‐term desmopressin treatment for central diabetes insipidus, and another dog had repeated hospitalizations to address persistent hypernatremia of undetermined cause. One dog needed long‐term desmopressin, developed iatrogenic hypoadrenocorticism secondary to mitotane administration, and had persistent hypernatremia.

## 4 DISCUSSION

Dogs with pituitary apoplexy have characteristic presenting complaints, exam abnormalities, and diagnostic findings, which facilitate diagnosis of this condition. In our study, 38% of dogs with pituitary apoplexy lacked evidence of an endocrinopathy before presentation. Presenting complaints included altered mentation, head pressing, signs of gastrointestinal dysfunction, inability to walk, collapse, ataxia, and restlessness. Upon examination, many dogs had an increased rectal temperature, changes in consciousness, cranial neuropathies, abnormalities in gait, and cervical discomfort. Magnetic resonance imaging showed a well‐defined intrasellar or suprasellar mass with acute intratumoral hemorrhage in all dogs. We did not identify any dogs with pituitary infarction. Fifteen of 26 dogs (58%) survived to discharge, of which 7 received medical management alone (median survival 143 days) and 8 received medications and radiation therapy (median survival 973 days).

The ages and weights of the dogs in our study are similar to other reports of dogs with pituitary tumors.^20^, ^21^ Median weights in these studies ranged from 21 to 23 kg and another case series of dogs with pituitary apoplexy^15^ reported a median weight of 27 kg, similar to the mean weight of 23 kg in our study. These studies do describe affected dogs that are smaller, as in our study. Dogs with apoplexy in these reports do appear larger on average than the cohorts in reports of dogs with pituitary‐dependent hyperadrenocorticism,^22^ and it is unclear if medium‐larger dogs are more predisposed to developing macroadenomas with or without apoplexy.

Clinical signs of dogs with pituitary masses include abnormal mentation, gait changes, cranial neuropathies, and pain.^21^, ^22^, ^23^ Similar findings (with the addition of vomiting) have been reported in dogs with pituitary apoplexy,^12^, ^13^, ^15^ and this was consistent with our study. Ptyalism or vomiting occurred in 52% of our cases, which we suspect is because of nausea in association with an acute change in intracranial pressure, although these signs might also be referable to vestibular dysfunction in some dogs. Seven of our dogs (27%) had signs of vestibular dysfunction (nystagmus, head tilt, vestibular ataxia), which did not include the dog with the presumptive brainstem meningioma. Two of these dogs had evidence of transtentorial herniation on MRI, which might have caused vestibular dysfunction, although another 7 dogs had transtentorial herniation without signs of vestibular dysfunction. Thus, transtentorial herniation appears to play a minor causative role in vestibular dysfunction in these dogs. Infarcts to the thalamus have been reported to cause signs of vestibular dysfunction in dogs,^24^ and it is possible that compression of the thalamus from suprasellar tumors can also cause vestibular dysfunction. A recent case series of dogs with pituitary apoplexy also described altered mentation and vestibular dysfunction as common clinical signs.^15^ However, in contrast to our study, seizures were a prominent clinical sign while postural or gait abnormalities and hyperpathia were uncommon and signs of gastrointestinal dysfunction and pyrexia were not mentioned.^15^

Almost half (46%) of the dogs in our study had an increased rectal temperature. In humans, 30% of patients with intracerebral hemorrhage will have a fever.^25^, ^26^ Theories for neurogenic fever include prostaglandin production, hemotoxic damage to the hypothalamus, and inhibition of blood supply to the midbrain, which might disinhibit thermogenesis.^27^ Fever has been shown to be negatively associated with a return to function and survival after intracerebral hemorrhage in humans.^28^

Although the mean systolic blood pressure of the dogs in our study was similar to reference values reported for clinically normal dogs in the literature, there was considerable variability in our cohort and 6 dogs had values that would be considered hypertensive or severely hypertensive according to consensus guidelines (>160 mm Hg).^29^ It is possible that pre‐existing hypertension precipitated or contributed to the hemorrhage associated with apoplexy in some of these cases. Systemic hypertension has been suggested to be a predisposing factor for pituitary apoplexy in humans,^30^, ^31^, ^32^ although several other reports, including a large, case‐control study found no association and has called this relationship into question.^33^, ^34^ The causative role of blood pressure in dogs is difficult to disentangle from a potential compensatory response to an acute intracranial increase in volume, situational hypertension because of anxiety, and other contributing comorbidities.

The severity of the signs of neurological dysfunction varied in the dogs in our study. This parallels reports of pituitary apoplexy in humans, in which presenting complaints range from headache to coma, and there are described grades of severity.^35^ Several factors might contribute to this varying severity, including the size of the pituitary tumor and associated hemorrhage, the rapidity of expansion of the mass lesion and the degree of perilesional edema.

Pituitary tumors are responsible for approximately 13% of all intracranial tumors in the dog and are subclassified as adenomas or carcinomas.^36^, ^37^ These neoplasms can be hormonally silent or functional. Functional tumors might lead to endocrine disorders, such as pituitary‐dependent hyperadrenocorticism, acromegaly, increased prolactin production, or the tumors can be plurihormonal.^23^, ^38^, ^39^, ^40^, ^41^ Furthermore, neoplastic tissue can lead to destruction of normal gland parenchyma and cause endocrinopathies associated with pituitary insufficiency, such as hypothyroidism or hypoadrenocorticism.^42^, ^43^ In our study, 62% of dogs with pituitary apoplexy had a concurrent endocrinopathy or signs consistent with an endocrine disorder (although 6 dogs [23%] were diagnosed with hypothyroidism, most of which were likely primary lymphocytic thyroiditis). In humans, symptoms of endocrine disturbances are often described in retrospect at the time of diagnosis.^32^, ^44^ Some of the dogs in our study had an acute onset or marked exacerbation of polyuria and polydipsia at the time of onset of signs of neurological disease. Another subset of our patients had new onset of hyposthenuria at the time of presentation. These findings suggest that an apoplectic event can cause complete or partial central diabetes insipidus in dogs. Central diabetes insipidus can also be encountered in humans with pituitary apoplexy, albeit in a small minority (<5%) of cases.^2^ Evaluation of pituitary function through hormonal testing was only performed in a subset of the dogs in our study and was inconsistent between cases, reflective of the study's retrospective nature. To better evaluate endocrine disturbances in dogs with pituitary apoplexy, investigation of hypothalamic‐pituitary‐adrenal axis function (eg, testing cortisol levels, adrenocorticotropic hormone stimulation, or dexamethasone suppression testing), thyroid function (eg, testing total T_4_ and thyroid‐stimulating hormone), and assessment of urinary concentration ability (eg, urinalysis, water deprivation testing, response to desmopressin acetate) should be considered.

While there have been MRI descriptions of pituitary tumors in dogs,^23^, ^45^, ^46^, ^47^, ^48^ previous descriptions of the MRI findings for pituitary apoplexy are limited.^14^, ^15^ Similar to a report including MRI findings in a cat with pituitary apoplexy and to most cases in the prior series in dogs,^15^, ^16^ our cases all had a suprasellar contrast‐enhancing lesion causing a mass effect, with susceptibility artifact on GRE/T2* or SWI imaging. Susceptibility artifacts on these sequences are useful for detecting hemosiderin, deoxyhemoglobin, ferritin, calcium, air or other paramagnetic or diamagnetic substances and are highly sensitive for the detection of hemorrhage.^49^, ^50^, ^51^, ^52^ Unlike the cat but similar to the dogs in the prior study, many of the dogs in our study had T1W and T2W hypo‐ to isointensity of the corresponding intralesional areas showing susceptibility artifact. There are distinctive forms of hemoglobin that occur during different ages of hemorrhage, which have unique magnetic properties and appearances on MRI.^53^ The appearance of these various stages of intracranial hemorrhage on MRI have been reported in humans, and hemorrhage that is iso‐ to hypointense on T1W and hypointense on T2W is consistent with deoxyhemoglobin that is expected in acute hemorrhage.^54^ Interestingly, 1 dog had susceptibility artifact extending into the ventricular system, with a corresponding pleocytosis. Together with the erythrophagocytosis noted on CSF examination, this is indicative of hemorrhage into the CSF of the ventricular system, which are reported previously.^15^ Ring enhancement after intravenous contrast administration is consistent with a neoplasm with associated subacute or acute hemorrhage.^55^ Therefore, MRI changes and the speed of onset of signs of neurological disease are consistent with the acuity of pituitary apoplexy in this cohort of dogs. Although all of the cases in our cohort had hemorrhagic apoplectic events, it is possible that infarction or ischemia of the pituitary gland may result in similar signs in dogs, as reported in humans.^2^, ^7^ In such scenarios, diffusion‐weighted imaging could improve detection of such lesions.

We describe CSF findings in a limited number of dogs with pituitary apoplexy, which were quite variable. Humans can also have variability in CSF results and there have been case reports of cytologically unremarkable CSF in pituitary apoplexy, or neutrophilic pleocytosis which can mimic infectious meningitis.^40^, ^56^, ^57^, ^58^, ^59^, ^60^, ^61^, ^62^, ^63^

All dogs received supportive care, but nearly half of the dogs (46%) did not survive to discharge. Dogs were treated at the discretion of the managing veterinarians and not all dogs received the same supportive treatment, which is a limitation of the retrospective nature of this study. Some owners elected humane euthanasia, given the clinical signs and the diagnosis of an intracranial tumor. However, many dogs that survived to discharge had a favorable outcome, with a median survival time of 973 days for dogs receiving medications and radiation therapy, and 143 days for those receiving medications alone. There might be a selection bias associated with the favorable outcome of dogs that received radiation therapy, as these dogs might have had less severe signs of neurological disease and might therefore have been considered more suitable radiation candidates than those dogs where radiation therapy was not pursued. Additionally, these owners might have been more committed and therefore more willing to tolerate neurological abnormalities associated with tumor growth or adverse radiation effects. The survival times of the dogs in our study that were discharged from the hospital are comparable to dogs with pituitary tumors in previous studies treated with either radiation therapy or supportive care,^20^, ^64^, ^65^, ^66^ and another case series of canine pituitary apoplexy reported survival times of 15.5 months in cases that were discharged from the hospital.^15^ This suggests that the long‐term prognosis can be favorable in dogs surviving an acute apoplectic event. Although statistically significant, the medians of the MGCS in dogs surviving and not surviving to hospital discharge only differed by 1 (16 vs 15), with overlapping ranges (10‐18 vs 9‐17) and thus must be interpreted cautiously when making therapeutic and prognostic decisions about individual dogs. Our analysis of potential risk factors and clinical signs that might be predictive of prognosis failed to show any associations with survival to discharge from the hospital. These analyses are likely influenced by the small numbers of dogs in each group and by owner decisions surrounding euthanasia. Administration of hyperosmolar compounds was associated with failure to survive to discharge. However, because of the study's retrospective nature, there was no standardization of dosing or administration and we suspect this association is primarily related to the use of these agents in cases with more severe clinical signs and advanced disease.

In humans, the decision to pursue decompressive surgery vs medical management, and the timeframe in which surgery should be performed, are controversial. In general, visual impairment and neurological deterioration are indications for surgery, as there is more likely to be visual improvement if the surgery is performed within 7 days of symptom onset.^67^ Transsphenoidal surgery has been well described in dogs as an effective treatment for pituitary‐dependent hyperadrenocorticism.^68^, ^69^, ^70^, ^71^, ^72^, ^73^ As in humans with pituitary apoplexy, this approach could be considered in dogs with this condition that are stable enough for general anesthesia.

This study has a number of limitations, many associated with its retrospective nature. Exclusion of dogs with clinical signs that had been present for longer than 1 week may have eliminated some cases that had an acute apoplectic event, but were not presented to our hospital within this initial time period. There was no standardization of the diagnostic tests performed, including coagulation testing, CSF analysis, endocrine evaluation or acquisition of MRI sequences. There was not a standard protocol for medications received, or radiation therapy dose and fractionation administered, which can complicate interpretation of the outcomes of these dogs. Survival was largely determined by owner decisions regarding euthanasia and not natural death. Finally, pituitary apoplexy was mainly identified based on the acuity of onset of neurological signs and MRI features consistent with acute hemorrhage, and only 2 dogs (8%) had histopathologic confirmation of this diagnosis.

This study provides a relatively large case series describing historical findings, clinical abnormalities, and outcomes in dogs with pituitary apoplexy. Furthermore, it expands upon limited published information describing the MRI and CSF findings, as well as outcomes of dogs with pituitary apoplexy. Dogs commonly have a historical endocrinopathy with an acute episode of altered mentation and signs of gastrointestinal dysfunction such as vomiting, ptyalism and anorexia. While many dogs are euthanized before discharge from the hospital, dogs that survive to discharge can have a favorable outcome.

## CONFLICT OF INTEREST DECLARATION

Authors declare no conflicts of interest.

## OFF‐LABEL ANTIMICROBIAL DECLARATION

Authors declare no off‐label use of antimicrobials.

## INSTITUTIONAL ANIMAL CARE AND USE COMMITTEE (IACUC) OR OTHER APPROVAL DECLARATION

Authors declare no IACUC or other approval was needed.

## HUMAN ETHICS APPROVAL DECLARATION

Authors declare human ethics approval was not needed for this study.

## Supporting information


**Table S1.** Cerebrospinal fluid analysis from 5 dogs with pituitary apoplexy. All samples were obtained from the cerebellomedullary cistern. Abbreviations: NCC: nucleated cell count, reference range 0‐4 cells/μL; RBCC: red blood cell count; TP: total protein, reference range 0‐25 mg/dL.

**Table S2.** RT plan details.
