# Supplementary material for: Presumed pituitary apoplexy in 26 dogs: Clinical findings, treatments, and outcomes
Source: J Vet Intern Med. 2023 Apr 21;37(3):1119–28. doi: 10.1111/jvim.16703 (PMC10229324; doi:10.1111/jvim.16703)
Supplement: Supplementary file 1 — Table S1. Cerebrospinal fluid analysis from 5 dogs with pituitary apoplexy. All samples were obtained from the cerebellomedullary cistern. Abbreviations: NCC: nucleated cell count, reference range 0‐4 cells/μL; RBCC: red blood cell count; TP: total protein, reference range 0‐25 mg/dL. [file JVIM-37-1119-s002.pdf]

	NCC	RBCC	TP	Cell differential	Interpretation
**Dog 1**	30	85	42.9	51% large mononuclear, 30% nondegenerate neutrophils, 17% small mononuclear, 2% eosinophils	Mononuclear pleocytosis
**Dog 2**	13	563	49.6	60% nondegenerate neutrophils, 35% large mononuclear cells, 5% small mononuclear cells	Neutrophilic pleocytosis with evidence of hemorrhage/ erythrophagocytosis
**Dog 3**	94	950	33.1	47% small mononuclear, 41% large mononuclear, 12% nondegenerate neutrophils	Mononuclear pleocytosis
**Dog 4**	2	348	42.8	41% small mononuclear, 32% nondegenerate neutrophils, 27% large mononuclear cells	Albuminocytologic dissociation with evidence of hemorrhage/ erythrophagocytosis
**Dog 5**	383	14,663	204	48% large mononuclear, 46% non-degenerate neutrophils, 6% small mononuclear	Mixed pleocytosis

Supplemental Table 1. Cerebrospinal fluid analysis from five dogs with pituitary apoplexy. All samples were obtained from the cerebellomedullary cistern. Abbreviations- NCC: nucleated cell count, reference range 0-4 cells/μl; RBCC: red blood cell count; TP: total protein, reference range 0-25 milligrams/dl.
